# A Peek into the Bacterial Microbiome of the Eurasian Red Squirrel (*Sciurus vulgaris*)

**DOI:** 10.3390/ani12050666

**Published:** 2022-03-07

**Authors:** Diana Ioana Olah, Emöke Páll, Constantin Cerbu, Sergiu Dan Zăblău, Gheorghiță Duca, Monica Ioana Suătean, Adrian Valentin Potârniche, Aurel Vasiu, Marina Spînu

**Affiliations:** 1 Faculty of Veterinary Medicine, University of Agricultural Sciences and Veterinary Medicine, 3-5 Mănăștur Street, 400372 Cluj-Napoca, Romania; constantin.cerbu@usamvcluj.ro (C.C.); sergiu.zablau@gmail.ro (S.D.Z.); suateanmonica@gmail.com (M.I.S.); adrian.potarniche@usamvcluj.ro (A.V.P.); aurel_vasiu@yahoo.com (A.V.); marina.spinu@gmail.com (M.S.); 2 Institute of Research and Development for Agro Montanology, Academy of Agricultural and Forestry Sciences “Gheorghe Ionescu Șișești”, Cristian, 557085 Sibiu, Romania; ghitaduca@yahoo.com

**Keywords:** *Sciurus vulgaris*, wild, captive, microbiome, environment

## Abstract

**Simple Summary:**

The Eurasian red squirrel can be found from Europe and Asia, but due to habitat destruction or fragmentation, other squirrel species, and infectious diseases, in some European countries, the species finds itself at the brink of extinction. In such areas, captive breeding and release into the wild may be part of the solution to repopulation. Captivity, nonetheless, has been shown to greatly influence the species’ microbiota relative to wild animals. Therefore, evaluation of the microbiota in both captive and wild squirrels could elucidate if special living conditions are needed in order to augment the survival rate of specimens reintroduced into the wild. Furthermore, the microflora profile of healthy red squirrels raised in captivity would support clinicians in addressing infectious disease episodes and also raise awareness of the zoonotic risk. Hence, this study documented the bacterial species carried by *S. vulgaris*, disclosing overall similarities and variability patterns of the microbiota identified in individuals from two different living environments. We predicted less diversity in the captive animals’ microbiota, due to the restrictive diet and unchanging living conditions, but a higher prevalence of zoonotic bacteria, due to the proximity of humans and of other domestic species. In this respect, samples (*n* = 100) were taken from five body regions of 20 red squirrels, both free-ranging and bred in captivity, processed by classical microbiology techniques and further identified by biochemical assay (VITEK^®^2 Compact System). A relatively poor bacterial community, comprising 62 bacterial strains belonging to 18 species and 8 different genera, was identified. Most of these microorganisms were reported for the first time in *S. vulgaris*. The results suggest that the resident aerobic microbiota of *S. vulgaris* does not differ significantly depending on living environments, neither in diversity, nor in quantity of the cultivable isolates.

**Abstract:**

*Sciurus vulgaris* (the Eurasian red squirrel) is native to Europe and Asia, but due to habitat destruction or fragmentation, interspecific competition, and infectious diseases, especially in European island areas the species finds itself at the brink of extinction. The repopulation of such bare habitats requires healthy squirrel specimens, either translocated from other wild habitats or reintroduced to the wilderness following captive breeding. Captivity, nonetheless, has shown an immense capacity to reshape the structure of wild species’ microbiota, adapting it to the less diverse diet and fewer environmental challenges. Therefore, assessing the differences between “wild” and “captive” microbiota in this species could elucidate if special living conditions are needed in order to augment the survival rate of specimens reintroduced into the wild. Furthermore, the microflora profile of the normal flora of healthy red squirrels raised in captivity could support clinicians in addressing infectious diseases episodes and also raise awareness on the zoonotic risk. Hence, this study aimed at documenting the bacterial species carried by *S. vulgaris*, disclosing overall similarities and variability patterns of the microbiota identified in individuals from two different living environments. We anticipated that the bacterial community would be less diverse in individuals raised in captivity, owing to their restrictive diet and to unchanging conditions in the enclosure. We also hypothesized that there would be a higher prevalence of zoonotic microorganisms in the captive animals, due to the proximity of humans and of other domestic species. To test this, samples (*n* = 100) were taken from five body regions of 20 red squirrels, both free-ranging and bred in captivity, processed by classical microbiology techniques, and further identified by biochemical assay (VITEK^®^2 Compact System). A relatively poor bacterial community, comprising 62 bacterial strains belonging to 18 species and 8 different genera, was identified. Most of these microorganisms were reported for the first time in *S. vulgaris*. With no discrimination between living environments, the highest prevalence (*p* < 0.001), was registered in *Staphylococcus sciuri* (60%; 12/20), followed by *Escherichia coli* (45%; 9/20) and *Bacillus cereus* (35%; 7/20). The results suggest unremarkable differences in diversity and richness of the resident aerobic microbiota of *S. vulgaris*, in relation to the living environment.

## 1. Introduction

In September 2019, on the IUCN official website [1], one could read: “More than 28,000 species are threatened with extinction. That is 27% of all assessed species.”. Less than a year and a half later, in February 2021, the figures changed: “More than 35,000 species are threatened with extinction. That is still 28% of all assessed species.” In September 2021, the number rose to 38,500, then to 40,000 species, in December 2021. Worldwide wildlife diversity is being lost at an alarming rate and many species go extinct even before they are discovered. In this gruesome setting, conservation efforts must address multiple facets, involving adjoint strategies, which include strong anti-deforestation and anti-poaching policies, feasible captive rearing, and reintroduction programs [2,3]. However, the groundwork for such strategies may only be laid with the preservation of equilibrium between the microorganisms, i.e., viruses, bacteria, archaea, fungi and protozoa, and their harboring host. The conformation of bacterial communities in animals, vertebrates or invertebrates is likely to be configured by multiple factors, such as host genotype [4,5,6,7], host diet [8], or breeding in laboratory conditions [9,10]. In recent decades, biologists have gained an appreciation for the roles played in the wildlife by the host-associated microbiome. These roles refer to the physiology, health and evolution of the host [11,12,13,14,15]. Some of the most surprising findings link microbial communities to the food-seeking behavior of the host. In other words, bacteria manipulate the procurement of resources required for optimal growth [16]. Moreover, microorganisms intervene in host behavior regulating certain social interactions in order to ensure their transmission from individual to individual [17]. This extraordinary interface between the host and its microbiota has entailed the unifying concept of the metaorganism [18].

The Eurasian red squirrel (*Sciurus vulgaris*) is native to Great Britain, continental Europe and Asia [19], known to be the only arboreal species endemic to Europe [20] until recently, when *S. meridionalis* was classified as a distinct species residing in Southern Italy [21] Its habitat expansion took place as a result of numerous relocations and it is listed as *least concern* within the Red List of IUCN (*International Union for Conservation of Nature*) [1]. However, in some of the European countries, red squirrels have become extinct because of habitat destruction and fragmentation [22]. In the UK and Ireland, interspecific competition with the non-native *S. carolinensis* (eastern gray squirrel) has caused the disappearance of red squirrels from large areas [20,23,24,25,26,27,28,29,30,31,32,33]. Moreover, the gray squirrel is incriminated in the effective dissemination of squirrel pox virus (SQPV), which causes epidemics and significant mortality in the red squirrel population, profoundly impacting the species distribution where the native and allogeneic species share habitats [25,31,32,33,34,35,36,37]. 

Studies on the microbiome of wild animals, such as giant panda [38], Tasmanian devil [39], howler monkey [13], red panda [40], capybara [41], koala [42] or American red squirrel (*Tamiasciurus hudsonicus*) [30,43,44] followed the growing awareness that the microbiota can mirror the health status of a population and also provide a measure of the impact of captivity on the survival of wild species. Undoubtably, bacterial infectious diseases result not only from the activity of pathogenic microorganisms, but also from the absence or unbalance of resident bacterial communities within the microflora. Captivity arguably exerts a major influence on the microbiome of wild species, reducing its abundance [39,45] and leading to this loss of equilibrium. Its various means of action include the transition from naturally rich to reduced food diversity, the decrease of microbial reservoirs in the environment, cohabitation with other animal species and the administration of antibiotics [46]. In most countries worldwide, or states in the USA, the captive breeding of Eurasian red squirrels is not prohibited by law. They have gained popularity as exotic pets, and thus the demand for trade has been rapidly growing. Haplessly, the ownership is not accompanied by the education required for proper nutrition and welfare of these animals. As a rough estimate provided by squirrel farm owners in Romania, one in three red squirrels, although born and raised in captivity, dies of unknown causes shortly after being relocated to its owner (unpublished data).

To our knowledge, the normal microbiota of the Eurasian red squirrel has not been studied to date. Only two of the 18 bacterial species identified in this study were found in other research focused on this animal. Insights into the natural bacterial inhabitants of *S. vulgaris* may have notable consequences, especially on successful repopulation attempts in areas where the species is on the verge of extinction. Furthermore, the study of wildlife microbial communities finds relevance beyond conservation issues. It targets the ‘One Health’ concept, which recognizes the interconnection between human, animal and environmental health. Recent studies show that animal hosts of zoonotic microorganisms proliferate in human-dominated ecosystems rather than less-disturbed areas, leading to the conclusion that biodiversity loss increases the risk of human exposure to multi-host infectious agents [47,48]. Therefore, detecting asymptomatic carriers, possible reservoirs of zoonotic agents [49], and knowledge of the manifestation of microorganisms in various niches is of utmost importance [50] 

The aim of this paper was to describe the resident aerobic microbiota in free-ranging and captivity-raised Eurasian red squirrels. We predicted that the bacterial community will be less diverse in individuals raised in captivity, owing to their restrictive diet and to unchanging conditions in the enclosure. We also hypothesized that the bacteriome diversity between the wild and controlled environment will differ, expressing a higher prevalence of zoonotic bacteria in the latter, due to the proximity of humans and of other domestic species. Furthermore, we trust that describing a pattern of the normal microflora of healthy red squirrels raised in captivity would support clinicians in addressing infectious diseases episodes.

## 2. Materials and Methods

### 2.1. Animals

For a comparative assessment of the microbiome between the living environments, the individuals included in the study originated from both the wild and from captivity. Fourteen of the 20 squirrels included in the study were bred in captivity on a squirrel farm located in Galati county, Romania. Three of the free-living squirrels were admitted to the New Pets Species Clinic within the Faculty of Veterinary Medicine of Cluj-Napoca with severe car collision trauma and the other three squirrels were immediate roadkill recovered from different regions of Transylvania, Romania. All the samples were obtained during the month of April of the same year. 

The squirrels were sexed based on the distance between the anus and urinary opening [51]. The age of the 9 females and 11 males was interpreted in the context of four age categories: adult, subadult/adult, subadult and juvenile, using the detailed criteria of Carrroll et al. [52] with reference to reproductive status and body mass. 

Free-ranging females with evidence of reproduction, such as the development of mammary glands, signs of active lactation (enlarged nipples/haloes of alopecia around the nipples) were considered adults. Females in which no signs of reproduction were noted but in which body weight reached approximately 300 g (indicative of sexual maturity) were introduced in the subadult/adult category. Those with no signs of reproductive activity, weighing less than 300 g and more than 150 g were classified as subadults, whereas juvenile females (kittens) weighed less than 150 g. The males were considered adults when testicles were developed, approximately 10 mm long and pigmented. In subadults, the scrotal testicles were small (<10 mm long) or abdominal, and the body mass exceeded 150 g. Males with a body weight under 150 g were classified as juvenile (Table 1). 

A lower age limit of 4–5 weeks was approximated in 4 of the squirrels, given that their eyes and ears had just opened. The weight of the animals ranged from 80 to 365 g. For five of the females, the owner was able to provide information on a previous parturition. These were considered adults without a thorough assessment, considering of utmost importance to reduce handling time, thus inducing less stress.

The squirrels raised on the farm were accommodated (alone or in pairs) in approximately 3 m long/3 m wide/2.5 m high aviaries, equipped with wooden nests and branches for the most accurate mimicry of natural conditions, and meeting very good hygiene conditions. The orientation of the enclosures allowed exposure to the sun, but also to wind and blizzards throughout the day. In the last 3 months prior to sampling, their diet consisted mostly of a mixture of oily fruits: nuts, hazelnuts and sunflower seeds, with the occasional apples and carrots mixture. The owner claimed he had never used antibiotic treatment, but administered a complex of vitamins, minerals and amino acids (Promotor L 47), 2 mL/1 L of water, for 7 days, every winter month. Only speculative information is known about the feeding habits in the wild specimens, but it can be assumed that there was a significant variation in their diet, supported by the plentiful coniferous, deciduous and mixed forests spreading across Transylvania. During winter and spring, squirrels would mainly feed on beechnut (*Fagus sylvatica* L.), spruce (*Picea abies* L.), acorns (*Quercus robur* L.), walnuts (*Juglans regia* L.), hazelnuts (*Corylus avellana* L.) pine (*Pinus sylvestris* L., *Pinus mugo* L., *Pinus cembra* L.), elm (*Ulmus glabra* Huds.), juniper (*Juniperus communis* L.) and larch (*Larix decidua* Mill.) seeds.

The captive animals were caught in their enclosures and immobilized safely by the owner. The average handling time per each animal approached 90 s. No harm was inflicted on the squirrels during the process, or any injury observed in the following days. Sampling was performed using sterile swabs previously soaked in 0.9% saline by lightly pressing the area for approximately 10 s to ensure contact of the swab with the sampled surface. The swabs were immediately inserted into glass tubes containing simple broth (Cooked Meat Medium, Oxoid, Hampshire, UK) and sealed tightly with plastic screw caps. Each tube was labelled with the number assigned to the sampled animal. Five body regions were sampled in each individual: the oral cavity, ear, interdigital, peri-genital and perianal areas, totaling 100 samples. At the time of sampling, none of the animals showed any symptoms of an infectious disease.

The samples obtained from the squirrel farm were placed into a plastic container, transported at 22 °C for approximately 5 h, and afterwards incubated, whereas the samples collected from the wild individuals were processed within 15 min after collection.

### 2.2. Standard Microbiological Investigation

In order to isolate as many bacterial species as possible, we started with the initial cultivation on the simplest classical culture media. Thus, the samples were primarily cultured on simple broth (Cooked Meat Medium, Oxoid, Hampshire, UK) for 24 h at 37 °C. Aliquots were afterwards plated on nutrient agar (Nutrient Agar, Tulip Diagnostics, Verna, India) and submitted to aerobic incubation, at 37 °C for 24 to 48 h. Subsequent to a primary culture evaluation, Gram stained smears were obtained from individual colonies with different morphologies. In case of overlapping or invasive growth of the colonies, secondary passages were performed on nutrient agar. Following microscopic examination, one colony of each strain was transferred to either MacConkey agar (MacConkey Agar, Tulip Diagnostics, Verna, India) for the selection of Gram-negative microorganisms, or Chapman agar (Chapman Stone Agar, HiMedia, Mumbai, India) for the selection of staphylococci. Samples showing no bacterial growth on selective media were re-inoculated on chromogenic and nutrient agar using the primary cultures (Chromogenic UTI Medium, Oxoid, Hampshire, UK). Twenty-four hour monocultures were prepared for biochemical identification by use of Vitek^®^2 Compact System. 

### 2.3. Biochemical Identification of Bacteria 

VITEK^®^2 (bioMérieux SA, F-69280 Marcy l’Etoile, France) is an automated microbial identification system. Special VITEK^®^2 GN cards (identifying 154 species of *Enterobacteriaceae* and a selected group of non-fermenting Gram-negative organisms), VITEK^®^2 GP (identifying 124 species of enterococci, streptococci, staphylococci, and a selected group of Gram-positive organisms) and VITEK^®^2 BCL (identifying 42 Gram-positive microorganisms of the *Bacillaceae* family) were used in the biochemical assay. The reactive cards have 64 wells, each containing an individual substrate. Substrates measure various metabolic activities such as acidification, alkalization, the use of carbon sources, enzymatic activities and resistance to inhibitory substances. 

### 2.4. Preservation of Microorganisms

Due to the gradual biochemical testing, some of the strains obtained were temporarily preserved. To this intent, one isolated colony from each of the monocultures was inoculated in 850 µL of sterile broth contained in 1.5 mL Eppendorf tubes. The samples were incubated for 24 h at 37 °C. Subsequently, bacterial growth was confirmedby broth turbidity, and 150 µL of sterile glycerol were added to each tube. All samples were maintained at −80 °C until use, and were revitalized when needed by inoculation on nutrient agar.

### 2.5. Statistical Analysis of the Data

The database and statistical analyses were performed in the Microsoft^®^ Office—Excel program and in the IBM^®^ SPSS (Version 19) statistical package. For the variables with normal distribution, the mean, standard deviation and standard error were calculated. According to these, the confidence intervals corresponding to a 95% confidence level were also obtained. The Pearson correlation was generated to verify the degree of dependence between normally distributed quantitative variables, i.e., to check for the association between the living environment and age, and the microbiota of squirrels. To determine whether the mean of a variable differed significantly from the population mean, we used the One-Sample t-test. The comparison of the means of two variables with equal and unequal variance was performed using the Student’s *t*-test (*p* < 0.05).

## 3. Results

Six of the 100 samples tested proved negative after cultivation. 

All microorganisms identified in this investigation fell into three different phyla. The dissimilarities with respect to different living environments of the animals were minimal. *Actinobacteria* had an overall representation of 1.69%, while *Firmicutes* were represented by 78.95% and 78.04%, *Proteobacteria* by 21.05% and 19.51%, of the identified bacteria in free and in captive animals, respectively.

A total of 62 bacterial strains belonging to 18 species and 8 different genera were identified. Two of the Gram-negative microorganisms could not be identified by the described protocol. 

With no discrimination between habitats, the highest prevalence (*p* < 0.001), was registered in *Staphylococcus sciuri* (60%; 12/20), followed by *Escherichia coli* (45%; 9/20) and *Bacillus cereus* (35%; 7/20) (Table 2). Nine (50%) of the identified species were isolated from single individuals.

Forty-four strains were found in the squirrels raised in captivity, while the free-living squirrels were carriers for 19 bacterial strains (Table 3).

Irrespective of the habitat, both the prevalence and the proportion of the total identified bacterial flora showed the highest values in the genus *Staphylococcus*, represented mostly by coagulase-negative species. Staphylococci were isolated with a frequency of 83.33% in free-living animals. At least one staphylococcal species was found in five of the squirrels, totaling 42.10% of the strains isolated from these individuals. A similar isolation frequency (85.71%) was also observed in captive animals; staphylococci were found in 12 of the 14 individuals. Overall, the genus *Staphylococcus* accounted for 37.29% of all identified species. *Staphylococcus sciuri* represented its genus with an overall prevalence of 60%. In free-living red squirrels, its isolation rate was of 83.33%, while in captive animals (7 positive out of 14), its prevalence was significantly lower (50%) (*p* < 0.01). 

An overall prevalence of 20% was noted for *Staphylococcus xylosus*. Only one strain (16.66%) was isolated from free-living, and three (21.42%) from the captive squirrels. *Staphylococcus capitis* and *Staphylococcus vitulinus* were only found in two wild individuals, equivalent to a prevalence of 7.14% and 5% for the wild group and all the animals, respectively. Two strains of *Staphylococcus lentus*, one strain of *Staphylococcus equorum* and one of *Staphylococcus hycus* were found solely in captive red squirrels, accounting for an isolation frequency of 14.28%, 7.14% and 7.14%, respectively. Referred to all studied animals, *S. lentus* showed a prevalence of 10%. The overall prevalence halved for the strains of *S. equorum* and *S. hycus*.

*Escherichia coli* was present in six of the 14 captive (42.86%) and in three of the six free-living specimens (50%) It represented 14.29% and 15.79% of the entire bacterial population isolated in the two groups. Bacteria of the genus *Enterobacter* (*E. cloacae* complex and *E. kobei*) were isolated from two of the captive-bred animals, accounting for 5% of the identified strains. 

Related to the entire identified bacterial population, the proportion of microorganisms belonging to genus *Enterococcus* was very similar between the two groups, 15.78% and 14.63% for the wild and for the captive, respectively. A prevalence of 50% of this genus was observed in the free-living and somewhat less, 42.85%, in the captive squirrels. Isolated from two of the specimens of each group, *E. faecalis* recorded a prevalence rate of 33.33% in squirrels with a free habitat and 14.28% from the captive environment. With only one identification in individuals from the wild and four in those raised in captivity, *E*. *faecium* showed a prevalence of 16.66% and 28.57%, respectively. *E. avium* was isolated from a single captive specimen, thus indicating a group prevalence of 7.14% and an overall prevalence of 5%.

The genus *Bacillus* was present at a ratio of 21% in the total bacterial population isolated from free-living, versus 19.51% in the bacterial population of captive squirrels. Their prevalence amounted to 66.55% for the free-living, and 57.14% for the captive animals, respectively. A prevalence of 16.66% was found in the free-living squirrels, for both *Bacillus* spp. and *B. subtilis*, with a single isolation from distinct individuals. *Bacillus cereus* was isolated from two specimens, with a prevalence of 33.33% in this group. In captive squirrels, the prevalence of *Bacillus* spp. was 21.42%, and that of *B. cereus* reached 35.71%.

*Aerococcus viridans* was isolated from two individuals bred in captivity, with an overall prevalence of 4.7%. *Pantoea agglomerans* was carried by a single individual in the captive group, with a prevalence of 7.14% in the respective group and 5% calculated for the entire batch included in the study. A similar situation was noted in the case of *Kocuria rosea*.

Of the total staphylococci identified, 63.63% could be isolated from the ear canal, 54.54% from the perigenital area, 45.45% from the perianal area, 40.90% from the interdigital area and only 22.72% from the oral cavity. The *Enterobacteriaceae* were present in proportions of 83.83%, 50%, 41.66%, 25% and 8.33% in the perianal area, in the perigenital area, in the oral cavity, in the interdigital area, and in the auricular canal, respectively. All identified enterococci could be cultured from samples collected from the perianal area, 75% from the oral cavity, 62.5% from the interdigital area, and 25% from both the auricular and perigenitalareas. *Bacillus* spp. was mostly isolated from interdigital samples (75%), followed by the ear canal (58.33%), the perianal area (33.33%), and the perigenital area (25%). No strains belonging to *Bacillus* were isolated from the oral cavity.

With regard to the sampling area, the highest prevalence for staphylococci was detected in the ear. As expected, members of the *Enterobacter* and *Enterococcus* were more often found in the perianal area, whereas *Bacillus* were mostly found in the interdigital area. Regardless of the living environment, the largest proportion of the isolates equally belonged to the orders *Enterobacteriales* and *Lactobacillales*, (42.10%), followed by the order *Bacillales* (12.19%) and *Actinomycetales*, which was poorly represented (5.26%). When placed into phyla, the most substantial proportion belonged to *Firmicutes* (68.41%), followed by 12.19% for *Proteobacteria*, and 5.26% for *Actinobacteria*. 

The cutaneous microbiome consisted mostly of members of *Bacillales* (70.73%), followed by *Lactobacillales* (19.51%) and *Enterobacteriales* (9.75%). Therefore, as opposed to the oral microbiome, *Proteobacteria* encompassed the majority of the bacterial population (70.73%), leaving *Firmicutes* with approximately one third of the entire skin resident flora (29.26%).

The bacteria of the perigenital microbiome were mostly (60%) members of the order *Bacillales*. *Enterobacteriales* and *Lactobacillales* contributed with 24% and 16%, respectively. The only representative of the *Proteobacteria*, *Bacillales* led the phylum, while *Firmicutes* showed a prevalence of 29.26%.

In spite of the proximity of the areas, the composition of the perianal microbiome notably differed from the microbiota of the perigenital area. The orders *Bacillales* and *Enterobacteriales* were found in similar proportions (33.33%). Bacteria of the order *Lactobacillales* accounted for 30% of the perianal microbiome, and *Actinomycetales* for only 3.33%. Furthermore, the *Firmicutes* constituted 63.33% of the perianal microbiome and the *Proteobacteria*, 33.33%, while the *Actinobacteria* accounted for only 3.33%.

## 4. Discussion

While interest in the microbiome is continuously growing, the volume of research addressing the role of the resident flora with reference to animal health is still limited. This scarcity remains in spite of a mounting concern for discovering the links between the microbiome and animal health, which would further explain the differences between ecosystems, species and populations [7]. As scarce information is to be found on the Eurasian red squirrel, discussions will generally refer to reports in other species of Sciuridae or in other species of wild, occasionally domestic mammals. 

The only mention of *Staphylococcus sciuri* in the Eurasian red squirrel leads to studies by Duff et al. [51,52], where it is described as secondary infection agent of poxviral lesions in the eye and gut. Kloos et al. [53] isolate strains of *S. sciuri* from the skin of healthy specimens of gray squirrels (*S. carolinensis*), flying squirrels (*Glaucomys volans*) and racoons (*Procyon lotor*). A 64% isolation frequency, markedly higher than in the findings of this study, was found for *S. xylosus* in *S. carolinensis*. Nagase et al. [54] isolate *S. xylosus* and *S. sciuri* with increased frequency from domestic animals. With respect to *S. sciuri,* a prevalence of 76.5% was found in horses, 83.3% in cows, 50% in dogs and 43% in laboratory mice, and 25% in pigs, 23.5% in horses, 36.7% in cows, 10% in dogs and 69.6% in laboratory mice for *S. xylosus*. A prevalence of 10% for *S. capitis* and *S. lentus* was described in dogs, and 12.5% for *S. capitis* in humans. By comparison, we found that red squirrels carried *S. sciuri* less often than cows and more often than dogs, and *S. lentus* had a similar abundance to that observed in dogs.

Almost similar results to those of the present investigation were provided by research on the prevalence of staphylococci in small wild mammals, which allocated *S. xylosus* a second place in isolation frequency (20.8%), after *S. succinus* (27.9%). *S. lentus* and *S. equorum* were also mentioned, with no reference to their frequency or prevalence [55]. *S. equorum* was identified for the first time in healthy horses [56]. Of little interest in animal research, *S. equorum* is not uncommonly mentioned as a natural fermentation agent of various meat preparations [48,49,50,51] or as a compound of starter cultures for dairy products [57]. By isolation of a single strain, this study also confirms the low prevalence of the microorganism in an animal species. It appears unlikely that it would have any potential as a resident member of the microbiota. 

Considering the isolation rate, only two of the seven species of staphylococci could be considered in the microbiota profile of the Eurasian red squirrel: *S. sciuri* and *S. xylosus*. Additionally, the results suggest that *S. sciuri* is more likely to be found on the skin of free-living rather than captive individuals. 

*E.coli* strains with low pathogenicity were previously isolated from *S. vulgaris* in a case of mortality due to cestode parasitism [58]. Its presence in the red squirrel has also been reported in a case of fatal candidiasis [59]. Strains of non-haemolytic *E. coli*, along with *S. aureus*, have been identified from various body sites of carcasses recovered from the wild in the UK [60]. Hemolytic strains of *E. coli* were found in three other cases of fatal necrotic enteritis, with the concomitant detection of Adenovirus type 1 [61]. However, all reports illustrated cases of disease, leaving this microorganism with an equivocal role in the pathological process. Adesiyun [62] reports non-hemolytic *E. coli* strains in 58% of 271 healthy wild animals tested, mainly common agouties (*Dasyprocta leporina*). The same study states a prevalence of 83% in the case of 175 wild animals bred in captivity. Ahmed et al. [63] identify *E. coli* in various species housed in a zoo in China, assessing a significant prevalence of 52.6%. The same research reports the identification of *Enterobacter cloacae*, with a presence of 4.7%. Even if the carriage level for *E. coli* was elevated overall, the results of our study do not reveal a significantly higher prevalence of *E. coli* in captive when compared to free-living animals. Interestingly enough, we could find the bacteria in both of the juvenile recovered from the wild, but only in one of the other four individuals, a subadult.

In an early study, Mundt [64] was able to confirm the presence of enterococci in 71% of 216 mammals tested, including *Tamias striatus* (striped squirrel), *S. carolinensis* (here referred to as *Tamias siurus*), and *Tamias hudsonicus* (red squirrel), suggesting an isolation pattern. He concluded that small animals, mainly herbivorous, were less populated by enterococci. The results indicated, however, that the gray squirrel served as a natural host of enterococci, unlike the smaller species of the family Sciuridae. We could find a 50% overall prevalence of these normal inhabitants of the gut, which allowed a different inference. Still, a relatively low carriage of this bacterium, along with the absence of other cultivable microorganisms (e.g., lactobacilli) with roles in balancing the gut environment is an intriguing finding. More so considering that even if the overall prevalence of *E. coli* and enterococci was similar, only one of the animals, a free-living juvenile, carried both microorganisms.

The members of the *Bacillus cereus* group are mainly commensal in many mammals, birds or reptiles, but can also exert a pathogenic role, causing intestinal (diarrhea) or extra-intestinal signs (urinary tract infections, meningitis, bacteremia) [65]. The use of *B. subtilis* as a probiotic, especially in broiler chickens, demonstrated its efficacy both as a modulator of the inflammatory response, improving performance by *Salmonella* colonization decrease, and as a heat stress reducing agent, asserting its beneficial role in the animal microbiota [66,67]. We could isolate *Bacillus* strains in more than half of the animals, which suggests that the skin of squirrels offers an adequate support for the colonization of this pathobiont. In perspective, it could either maintain a healthy relation to its hosts, or readily infect skin wounds, as reported in human traumatic patients [68].

*Aerococcus* spp. are Gram-positive, catalase-negative, aerobic, facultative anaerobic germs, isolated for the first time from samples collected from the air and dust of inhabited rooms [69]. *A. viridans* is not considered a commensal of animal organisms, since it was isolated in cases of subclinical mastitis [70], and was associated with sepsis in immunodeficient mice [71]. Martin et al. [72] isolated the bacterium from lesions due to arthritis, meningitis and pneumonia in swine. In the two adult and subadult captive squirrels housed independently, the microorganism was isolated from the mouth, genital, anal and interdigital areas, but not from the ear. Given the opportune situation, this intensive colonization of the body may stand as requisite for a future infection. Although it is known for its ability to cause serious infections in humans, *A. viridans* could pose a threat to caretakers, owners or medical practitioners [73,74]. Still, its presence holds no statistical relevance in the microbiome profile.

*Pantoea agglomerans*, an enterobacterium recently separated from the genus *Enterobacter*, is considered a ubiquitous environmental microorganism, symbiont and sometimes pathogen of plants. The microorganism has been incriminated in cases of hemorrhagic disease in dolphin fish (*Coryphaena hippurus*) [75] and of equine abortion [76] (It is more often reported as an opportunistic agent, causing serious infections in humans [77]). There was a single isolation of *P. agglomerans* form the ear of an adult wild squirrel, which we considered a random carrier.

*K. rosea* is another seemingly harmless microorganism, a normal inhabitant of the skin and mucous membranes in humans and animals, frequently isolated from the environment. Species of the genus *Kocuria* are cocoid-shaped actinobacteria, Gram-positive, coagulase-negative, members of the *Micrococcaceae* family. For many years regarded as a laboratory contaminant, it gradually earns its place among the bacteria of pathogenic potential [72]. By reason of its insignificant prevalence (oral and perianal single carriage in a captive juvenile), its relevance in the microbiota of the studied red squirrels cannot be discussed. 

Recent studies which imply bacterial genomic DNA extraction and 16S rRNA gene sequencing were able to establish well-documented relationships between bacterial families, orders, and phyla in some wild mammal species. Of the 182 families and 21 bacterial phyla found in cecum in adult capybara (*Hydrochoerus hydrochaeris*), García Amado et al. [44] reported the majority of isolated bacteria were distributed in only five phyla: *Firmicutes*, *Proteobacteria*, *Bacteroidetes*, *Actinobacteria* and *Sphirochaetes*.

A study on two free-ranging koalas (*Phascolarctos cinereus*) showed a digestive microbiome mostly belonging to the *Bacteroidetes* and *Firmicutes* [78]. The oral microbiome of other two koalas was dominated by *Proteobacteria* and *Bacteroidetes*, while the rest largely belonged to the *Firmicutes* [42]. The preponderance of *Proteobacteria*, *Firmicutes* and *Bacteroidetes* was also emphasized after investigating the oral microbiota of 14 specimens of American red squirrels (*Tamiasciurus hudsonicus*). Their intestinal microbiome was dominated mainly by *Firmicutes*, *Proteobacteria* and *Bacteroidetes*, while the prevalent genera were *Prevotella* and *Coprococcus* [30]. In comparison, Ren et al. [43] reported the highest prevalence for *Firmicutes*, followed by *Bacteroidetes* and *Proteobacteria*. In the oral microbiota of *Macropus eugenii* kangaroos, results showed the presence of 53 different phenotypes belonging to *Firmicutes*, *Proteobacteria*, *Actinobacteria* and *Bacteroidetes* [79]. Alfano et al. [42] detected different proportions of the bacterial phyla in the rectal microbiome of koalas, in the following hierarchy: *Bacteroidetes*, *Firmicutes*, *Proteobacteria* and *Actinobacteri*. The study of the fecal microbiome of 16 free-living and six captive red pandas outlined the dominance of *Firmicutes* in bacterial populations carried by captive animals. The microbiome of wild subjects was more diverse and more evenly distributed among *Firmicutes*, *Proteobacteria*, and *Bacteroidetes* [40]. The oral and perianal microflora of both captive and free-living squirrels investigated in this study was dominated by *Firmicutes* and followed by *Proteobacteria*, but members of *Bacteroidetes* were completely absent. 

The phylum *Bacterioidetes* comprises Gram-negative, heterotrophic, unsporulated, aerobic or anaerobic rod-shaped bacteria, included in seven classes, of which *Bacteroidia*, *Cytophagia* and *Flavobacteria* are the best represented. Their presence is demonstrated in all ecosystems, from the depths of the oceans to deserts [80]. *Bacterioidetes* abundance in the intestinal microbiome has been positively associated with a diet rich in animal protein and fat, and negatively associated with high fiber intake [81]. The taxa included here are essential in degrading high molecular weight organic matter, e.g., proteins and carbohydrates and, to a lesser degree, various plant toxins [13,82]. Their part is all the more important in the microbiota of captivity-raised squirrels, which are frequently fed large quantities of carbohydrate-dense fruits or seeds known to contain harmful constituents. This includes the chemical compound cyanide, found in acorns, almonds, apple and pear seeds, apricot kernels, cherries, peaches and plum pits, and the fungicidal toxin persin, present in avocado skin. Improper diet also leads to obesity, one of the common and undebated problems of captivity-raised squirrels. An increase in abundance of *Firmicutes* and decrease in *Bacteroidetes* was seen in the gut microbiome of laboratory mice fed a high-fat high-sugar diet [82,83]. Although it is possible that the two biochemically unidentified Gram-negative bacteria isolated in this study belong to the *Bacterioidetes* phylum, either the marked paucity or the total absence of representatives is worrisome. However, it cannot be ruled out that the classical laboratory cultivation might not provide the growth requirements of strains inhabiting the intestinal tract of this species and belonging to the *Bacteroidetes*, just as it was once accepted that 80% of all bacterial species colonizing the human intestinal tract would not survive elsewhere [84]. Moreover, the research focused on the cultivation and identification of aerobic bacteria, and a considerable proportion of the members of this group are anaerobic microorganisms.

Studies evaluating microbiota of free-ranging and captive animals such as the black howler monkeys, Tasmanian devils and Andean bears have reported loss of diversity with captivity [13,39,85]. This would increase the susceptibility of captive individuals to infectious, inflammatory, degenerative or metabolic diseases [83,86,87]. However, the diversity remained consistent in bovids, giraffes, anteaters and aardvarks and it even increased in rhinoceros [88].When comparing the global microflora isolated from the two groups with different living environments by the Student’s t-test and assuming unequal variances, a statistically significant difference was observed between the averages of the identified bacterial populations (*p* < 0.01). The captive-bred squirrels were carriers of a larger number of bacteria and of a more diverse bacterial population. Nevertheless, if the results were correlated with more than double the number of animals raised in captivity, it was observed that the difference converged to zeo. As such, the number of bacterial isolates did not differ significantly between the two living environments, and the association between living environment and the presence of certain bacteria in the squirrels could not be certified. One of the reasons possibly underlying this finding concerns the housing of captivity-bred animals, as they were constantly exposed to the environmental conditions, with no protection from the influence of the various factors able to shape the microbiome of any living species. Another cause of the relative similarity in the microbiota of the two groups may be found in the varied diet offered to captive squirrels. In addition, the limited contact with caretakers, which consisted of sanitation of the shelter and food placement, did not enable the interspecific transmission of multi-host microorganisms. 

In contrast to findings of previous studies on wild animal microbiota, no role could be found for the age of the squirrels in variations of the microbiota (Pearson correlation–very weak; r ∈ [0–0.2] for each of the bacterial strains in relation to age category). The bacterial diversity and abundance were lower in juvenile than in older individuals in species like dugong, the white-cheeked gibbon and the Namibian cheetah [87,89,90]. In human subjects, this has been attributed to the reduced diversity of their diet consumed during early life [91]. By the age of about six weeks, the microbiota of these animals was stable and closely resembled that carried by adults.

Various microorganisms naturally colonizing the animal host and once considered harmless were subsequently found to produce prominent infections in humans. This is the case in multiresistant staphylococci [92], which can serve as reservoirs of antimicrobial resistance genes [93], cause surgical wound infections [94], urinary tract infections [95], sinusitis [96], and even pericarditis [97] and septicemia [98]. The havoc *E. coli* generates through its intricate pathogenetic mechanism is well documented [99]. Owing to their remarkable resistance to antimicrobials, disinfectants and harsh environmental conditions, *E. faecalis* and *E. faecium* give rise to unmanageable hospital outbreaks [100]. From food-borne [101], to skin, bone and joint infections [102] to pulmonary anthrax-like disease [103] and even death [102], *B. cereus* still tools its way to unlimited pathogenic power. These bacteria were also found in red squirrels subject to this research, regardless of their habitats, thus posing risks to the species, contacts and environment.

These results must be interpreted with caution and a number of limitations borne in mind. The simple isolation protocol of the microorganisms did not allow the growth of fastidious and/or anaerobic microorganisms. Biochemical assays can misidentify closely related bacteria or fail to identify the ones not included in their database. Unfortunately, financial resources did not allow the further application of molecular analysis. The non-uniform batches did not enable a proper comparison of the wild and captive microbiota. The low sample size may have reduced the diversity of the isolates and the reliability of the study, impairing the extrapolation of results. 

## 5. Conclusions

These results refute our hypothesis and suggest that there is a minor variation of microorganisms in the microbiome depending on the living environment of the red squirrel. This minor dissimilarity was in favor of the captive squirrels, and it may be connected to: (1) the housing conditions, which allow the captive animals to come in contact with bacterial strains ubiquitous in the external environment and (2) the well-balanced diet provided to captive squirrels, resembling the natural living conditions of this species. The presence of a relatively reduced number of microorganisms may be due to the special cultivation requirements of some strains, especially of those that colonize the gastrointestinal tract. Regardless of the environment, the most prevalent microorganisms, such as *Staphylococcus* and *Escherichia*, are recognized pathogens in a wide variety of hosts other than the red squirrel. These findings may be relevant for One Health professionals, owners and breeders. 

Because of the small sample size, this study is best viewed solely as a pilot study. The small size of the population studied and uneven distribution of individuals in the groups (free-ranging vs. raised in captivity) prevented reliable comparisons and correlations between the microbiota in connection to the two living environments. A more complete picture of the bacteriome of this species requires further research, both in captive and free-living red squirrels.

## Figures and Tables

**Table 1:** Characteristics of the squirrels sampled for the study.

No.	Living Environment	Gender	Age Category	Weight (Grams)
1	FR	M	A	323
2	FR	M	A	277
3	FR	M	J	73
4	FR	M	J	104
5	FR	F	SA/A	223
6	FR	F	SA	182
7	BC	M	SA	275
8	BC	F	A	320
9	BC	M	SA	202
10	BC	F	A	307
11	BC	M	A	383
12	BC	F	A	292
13	BC	F	SA/A	311
14	BC	M	A	365
15	BC	F	J	97
16	BC	F	J	80
17	BC	F	A	298
18	BC	M	A	337
19	BC	F	A	274
20	BC	M	J	92

M = male; F = female; A = adult; SA = subadult; SA/A = subadult/adult; J = juvenile; FR = free-ranging; BC = bred in captivity.

**Table 2:** The prevalence of the identified bacterial strains.

One-Sample *t*-Test					
Microorganisms		Test Value = 0			
	Proportion%	t	Significance Threshold (*p*)	Average Difference	95% Confidence Interval
*S. sciuri*	19.67	5.339	0.000	**0.600**	0.36–0.84
*E. coli*	14.75	3.943	0.001	**0.450**	0.21–0.69
*E. faecalis* *	6.55	2.517	0.021	**0.250**	0.04–0.46
*S. xylosus* *	6.55	2.179	0.042	**0.200**	0.01–0.39
*E. faecium* *	8.19	2.517	0.021	**0.250**	0.04–0.46
*S. lentus* *	3.27	1.453	0.163	0.100	−0.04–0.24
*E. cloacae* complex *	1.69	1.000	0.330	0.050	−0.05–0.15
*S. equorum* *	1.69	1.000	0.330	0.050	−0.05–0.15
*S. capitis* *	1.69	1.000	0.330	0.050	−0.05–0.15
*E. avium* *	1.69	1.000	0.330	0.050	−0.05–0.15
*Bacillus* spp.	6.55	2.179	0.042	**0.200**	0.01–0.39
*B. cereus* *	11.47	3.199	0.005	**0.350**	0.12–0.58
*A. viridans* *	3.27	1.453	0.163	0.100	−0.04–0.24
*B. subtilis* *	1.69	1.000	0.330	0.050	−0.05–0.15
*P. agglomerans* *	1.69	1.000	0.330	0.050	−0.05–0.15
*S. vitulinus* *	1.69	1.000	0.330	0.050	−0.05–0.15
*K rosea* *	1.69	1.000	0.330	0.050	−0.05–0.15
*E. kobei* *	1.69	1.000	0.330	0.050	−0.05–0.15
*S. hycus* *	1.69	1.000	0.330	0.050	−0.05–0.15
GN UI bacilli **	3.27	1.453	0.163	0.100	−0.04–0.24

Bolded average differences are statistically significant. * Species identified for the first time in *S. vulgaris.* ** Gram-negative unidentified bacilli.

**Table 3:** The prevalence of isolated bacteria corresponding to different living environments.

Bacterial Strains	Habitat	
	Wilderness	Captivity
	N (%)	N (%)
*S. sciuri*	5 (41.67)	7 (58.33)
*E. coli*	3 (33.33)	6 (66.67)
*E. faecalis*	2 (50)	2 (50)
*S. xylosus*	1 (25)	3 (75)
*E. faecium*	1 (20)	4 (80)
*S. lentus*		2 (100)
*E. cloacae* complex		1 (100)
*S. equorum*		1 (100)
*S. capitis*	1 (100)	
*E. avium*		1 (100)
*Bacillus* spp.	1 (25)	3 (75)
*B. cereus*	2 (28.57)	5 (71.43)
*A. viridans*		2 (100)
*B. subtilis*	1 (100)	
*P. agglomerans*	1 (100)	
*S. vitulinus*	1 (100)	
*K. rosea*		1 (100)
*E. kobei*		1 (100)
*S. hycus*		1 (100)
Unidentified Gram-negative bacilli		2 (100)

## Data Availability

The data presented in this study are available on request from the corresponding author.
